# Incorporating Prior Knowledge of Principal Components in Genomic Prediction

**DOI:** 10.3389/fgene.2018.00289

**Published:** 2018-08-02

**Authors:** Sayed M. Hosseini-Vardanjani, Mohammad M. Shariati, Hossein Moradi Shahrebabak, Mojtaba Tahmoorespur

**Affiliations:** ^1^Department of Animal Science, Ferdowsi University of Mashhad, Mashhad, Iran; ^2^Department of Animal Science, University College of Agriculture and Natural Resources, University of Tehran, Tehran, Iran

**Keywords:** genomic selection, statistical models, variable selection, principal component analysis, accuracy

## Abstract

Genomic prediction using a large number of markers is challenging, due to the curse of dimensionality as well as multicollinearity arising from linkage disequilibrium between markers. Several methods have been proposed to solve these problems such as Principal Component Analysis (PCA) that is commonly used to reduce the dimension of predictor variables by generating orthogonal variables. Usually, the knowledge from PCA is incorporated in genomic prediction, assuming equal variance for the PCs or a variance proportional to the eigenvalues, both treat variances as fixed. Here, three prior distributions including normal, scaled-t and double exponential were assumed for PC effects in a Bayesian framework with a subset of PCs. These developed PCR models (dPCRm) were compared to routine genomic prediction models (RGPM) i.e., ridge and Bayesian ridge regression, BayesA, BayesB, and PC regression with a subset of PCs but PC variances predefined as proportional to the eigenvalues (PCR-Eigen). The performance of methods was compared by simulating a single trait with heritability of 0.25 on a genome consisted of 3,000 SNPs on three chromosomes and QTL numbers of 15, 60, and 105. After 500 generations of random mating as the historical population, a population was isolated and mated for another 15 generations. The generations 8 and 9 of recent population were used as the reference population and the next six generations as validation populations. The accuracy and bias of predictions were evaluated within the reference population, and each of validation populations. The accuracies of dPCRm were similar to RGPM (0.536 to 0.664 vs. 0.542 to 0.671), and higher than the accuracies of PCR-Eigen (0.504 to 0.641) within reference population over different QTL numbers. Decline in accuracies in validation populations were from 0.633 to 0.310, 0.639 to 0.313, and 0.617 to 0.298 using dPCRm, RGPM and PCR-Eigen, respectively. Prediction biases of dPCRm and RGPM were similar and always much less than biases of PCR-Eigen. In conclusion assuming PC variances as random variables via prior specification yielded higher accuracy than PCR-Eigen and same accuracy as RGPM, while fewer predictors were used.

## Introduction

Advances in high-throughput genotyping technology allow the collection and storage of thousands to millions of SNP markers from many livestock species (Van Tassell et al., 2008; Matukumalli et al., 2009). These genotyped markers are a rich source of information, which can greatly enhance the performance of selection process for the genetic improvement of livestock. The information embedded in genotyped markers can be efficiently extracted by accurate models that can describe and predict the genetic merit of animals.

In genomic selection, relatively small number of phenotypes or pseudo-phenotypes are regressed on a large number of marker variables, simultaneously (Meuwissen et al., 2001). Regressing phenotypes on many marker variables raises several statistical and computational issues, such as how to confront the so-called “curse of dimensionality” as well as the complexity of a genetic mechanism that can involve various types and orders of interactions (Pérez and de Los Campos, 2014). It is expected that such data imbalance between markers and phenotypes still represents the main constraint on the implementation of genomic selection, especially for breeds other than Holstein (Pintus et al., 2012). Besides the “curse of dimensionality,” another challenging problem is multicollinearity arising from inter-correlation of marker genotype due to linkage disequilibrium (Long et al., 2011). These statistical challenges have been considered before, and several methods, such as partial least square regression (Wold, 1985), and principal component analysis (Peason, 1901; Hotelling, 1933) have been proposed to reduce the dimensionality of a data set.

Principal Component Analysis (PCA) belongs to the general framework of multivariate analysis and is one of the classical data analysis tools for dimension reduction (Jolliffe, 2002). In PCA, we seek to reduce the dimensionality of an m-dimensional data vector to a smaller p-dimensional vector, where p<<m, which represents an embedding of the data in a lower dimensional space. This technique is a widely used tool in genome-wide association studies to reduce the number of correlated traits (Bolormaa et al., 2010), to trace the respective contributions of population structure and LD between single nucleotide polymorphisms (NP) and quantitative trait locus (QTL) in the accuracy of genomic predictions (Price et al., 2006; Daetwyler et al., 2012), and for genomic prediction (Solberg et al., 2009a; Pintus et al., 2012). Macciotta et al. (2010) applied PCA approach to a PC-BLUP genomic prediction using eigenvalues as prior PC variances and conclude that results were better than the previous assumption of equal variance for PC effects in Solberg et al. (2009a), since the assumption of one single variance for all PC effects could be unrealistic.

In practice, however, when some principal components are excluded from the analysis by a selective criterion the sum of eigenvalues in remaining principal components is not equal to one. So the estimated variance will be smaller than the original variance, which makes scaling inevitable. In addition, when some variables are excluded from the analysis the ranking of the remaining variables is not necessarily the same as before. Exploiting this information may enhance the accuracy of predictions in a statistical analysis. Unfortunately, neither assumption of equal variance nor the assumption of eigenvalues as the prior variance for the predictors would accommodate such information as both techniques consider the variance(s) of predictors as fixed quantities.

External information can be incorporated into the regression on principal components through a Bayesian analysis, in which all parameters are considered as random effects with a probability density function that describes their contributions. Bayesian methods are common in genomic prediction with markers; however, genomic prediction models with PCs using realistic prior specification for PC scores have not been investigated yet. So, the aim of this study was to investigate the performance of a new Bayesian technique for genomic prediction with principal components to improve the accuracy of predictions by incorporating prior knowledge to PC effects and their variances.

## Materials and methods

### Simulation genome and population

Data were simulated using the QMSim software package (Sargolzaei and Schenkel, 2009) in 10 replicates for each scenario as follows. A single trait with phenotypic variance of one and heritability of 0.25 were produced. The genome consisted of 3 chromosomes, each one Morgan long. In total, 3,000 bi-allelic marker loci (single nucleotide polymorphism; SNP) and 105, 60, and 15 multi-allelic QTL were simulated on the genome. Markers and QTL positions were randomly selected across the genome. Mutation rate was set to 1 × 10^−3^ for markers and 1 × 10^−5^ for QTL, respectively. All genetic variance was due to additive QTL effects, which were randomly sampled from a gamma distribution with shape parameter 0.4. Phenotypes were generated for both sexes by adding random residuals from independent distributions$\sim N(0,\;I\sigma_{e}^{2})$ to the sum of QTL effects, therefore, no sex difference was simulated.

In order to achieve mutation-drift balance, historical generation was started with 400 females and 20 males and continued as follows: During 100 generation of random mating, the size of population increased to 1,000 animals. The population with the same size randomly mated for 400 more generations. The number of male animals in the last generation increased to 70. From generation 500, 35 males and 455 females were randomly selected as the generation zero and were mated for 15 generations. The mating design in the last 15 generations was also random, but to mimic a situation with selection, male and females were selected from the best animals with high breeding values of previous generation. Generations 8 and 9 were selected as training animals and generations 10 to 15 as selection candidates.

### Statistical computation

In this research two different groups of models were studied, SNP and PC based models, that used SNPs and PC scores as independent variables, respectively.

The general model for the record of individual *i*, *y*_*i*_, with observed marker genotype *j* labeled **Z**_*ij*_in the first group of models was:

(1) $$\begin{array}{r} y_{i}=\mu +sex_{k}+\sum_{j=1}^{m}Z_{ij}b_{j}+e_{i}, \end{array}$$

Where μ is the overall mean, *sex*_*k*_ is the effect of *k*th sex, *b*_*j*_ is the effect of marker genotype *j*, and there are *m* markers, and *e*_*i*_ is residual. In matrix notation the model is written as:

(2) $$\begin{array}{r} y=Xs+Zb+e, \end{array}$$

Where ***y*** a column vector of records of length n, ***s*** is a vector of fixed effects, ***X*** is incidence matrix that relates observations to fixed effects and ***Z*** is an n × m matrix with elements ***Z***_*ij*_ represented the marker genotype coded as −1, 0, and 1. A SNP genotype was removed if the SNP minor allele frequency (MAF) was less than 0.01 and if it deviated greatly from Hardy–Weinberg equilibrium (*P* < 1 × 10^−5^).

The alternative methods in the first group includes Bayesian Ridge regression (Bayes-Ridge), BayesA, BayesB, which differed in the prior used for ***b*** that are well known and most commonly used in genomic selection (Meuwissen et al., 2001; Habier et al., 2007, 2011). In Bayes-Ridge, the column vector of SNP effects is assumed to have the normal distribution $b\left(0,\;I\sigma_{b}^{2}\right)$, where $\sigma_{b}^{2}$ is the prior variance of the SNP effect sampling from scaled inverse chi-square prior with scale parameter $S_{b}^{2}$ and ν_*b*_ degrees of freedom as hyper-parameters. In BayesA, the marginal distribution of marker effects is a scaled-t density. But, it was shown that this is equivalent to assuming that the marker effect at locus j has a univariate normal with a null mean and unknown locus-specific variance $\sigma_{bj}^{2}$ (Gianola et al., 2009). In BayesB marker effects are assigned IID priors that are mixtures of a point of mass at zero and a slab that is a scaled-t density. The slab is structured as BayesA by introducing an additional parameter π represents the prior proportion of zero effects that is treated as unknown as previously emphasized that shrinkage of SNP effects is affected by π, and thus should be treated as an unknown being inferred from the data (Habier et al., 2011), therefore, it is assigned a Beta prior with the default hyper-parameters set by BGLR (Pérez and de Los Campos, 2014). In all the Bayesian models a flat prior (Sorensen and Gianola, 2002) is used for fixed effects and conditional on the residual variance,${\;\sigma }_{e}^{2}$, a normal distribution with null mean and co-variance matrix $\text{I}\sigma_{e}^{2}$ is used for the vector of residuals. Further, $\sigma_{e}^{2}$ is treated as an unknown with a scaled inverse chi-square prior. Variance hyper-parameters, i.e., scale and degrees of freedom, were set as BGLR defaults such that a proper but weakly informative prior distribution is postulated (Pérez and de Los Campos, 2014). Variance components with weakly informative priors will be less dependent on the prior setting and their posterior distribution will be dominated by the data (Sorensen and Gianola, 2002). The fourth model in the first group of models was Ridge-regression BLUP (Ridge-R) which used $\frac{\sigma_{a}^{2}}{m}$ as a variance of SNP effects. The mixed model equations of Ridge-R were simply solved in a non-Bayesian manner by Cholesky decomposition in R.

The second group of models using PC scores as the predictor variable were performed as follows. PCA was implemented on the correlation matrix of marker genotype (**W**_**m×m**_) as below (Janss et al., 2012):

(3) $$\begin{array}{r} W=UDU^{\text{T}}=\sum_{j=1}^{m}\lambda_{j}U_{j}U_{j}^{\text{T}}, \end{array}$$

Where ***U* = [*U***_**1**_**,*U***_**2**_**, …,*U***_***m***_**]** of order *m* × *m* is the matrix of eigenvectors of **W** with the ***U*_*j*_** represent the *j*th column, and ***D*** is a diagonal matrix with elements equal to the eigenvalues λ_1_, λ_2_, …, λ_*m*_ associated with the *m* eigenvectors. Properties of the eigenvalues and eigenvectors are λ_1_ > λ_2_ > …> λ_*m*_ and $U_{j}U_{j}^{T}=U_{j}^{T}U_{j}=I$, repectively. The choice of the number of PCs to be retained is arbitrary and several methods have been proposed (Jolliffe, 2002). In this study, we retain a *k* number of components until the cumulative variance reaching to 0.999 and then PC score were calculated for animals as:

(4) $$\begin{array}{r} {Z_{pc}=Z}_{x\times m}\%*\%{\;U}_{m\times k}, \end{array}$$

Where *x* denotes the number of individuals of training population or each of selection candidate sets. This **Z**_**pc**_ matrix was replacement as the incidence matrix for different PC based models as follows:

(5) $$\begin{array}{r} y=Xs+Z_{pc}b_{pc}+e, \end{array}$$

The alternative PC based methods hereinafter differ only in the prior used for the vector of predictor variables,***b***_***pc***_, and their variance. Principal component regression with eigenvalue as prior variance of predictor variable (PCR-Eigen) assumes that contribution of each PC score is proportional to their eigenvalues and therefore variances of each PC score was ccalculated as $\sigma_{pcj}^{2}$ = $\sigma_{a}^{2}\lambda_{j}$, where $\sigma_{a}^{2}$ is the additive genetic variance (Macciotta et al., 2010). It's BLUP mixed model equations were constructed and solved in R using Cholesky decomposition. In Bayesian principal component regression with normal distribution (PCR-Normal), regression coefficients are assigned to IID normal distributions, with mean zero and variance $\sigma_{pc}^{2}$ that the variance parameter is assigned a scaled-inverse Chi-squared density, with parameters *df*_*pc*_ and *S*_*pc*_. Bayesian principal component regression with t-density (PCR-t) was performed with assuming a scaled-t density as marginal distribution of predictor effects with parameters *df*_*pc*_ and *S*_*pc*_. However, as discussed in Gianola et al. (2009), this density is implemented as a univariate normal with null mean and unknown locus-specific variance $\sigma_{pcj}^{2}$ and the variance parameter is assigned an IID scaled-inverse Chi-squared density, with parameters *df*_*pc*_ and *S*_*pc*_. A double exponential distribution was assumed as marginal distribution of PC score in Bayesian principal component regression with a LASSO density (PCR-Lasso). The prior of double exponential distribution can be represented as an infinite mixture of scaled normal distributions (Park and Casella, 2008). Predictor effects are assigned independent normal densities with null mean and maker-specific variance parameter $\tau_{pcj}^{2}$ $\times \sigma_{\varepsilon }^{2},$ in the first. Second, $\tau_{pcj}^{2}$ are assigned IID exponential densities with rate parameter γ^2^/2. Finally γ^2^ assigned to a Gamma prior. A Gibbs-Sampling algorithm was used to estimate PC effects and their variance simultaneously.

### Predictive ability

Different models were compared on how accurately they predict the true breeding values of animals. The correlation between genomic estimated breeding values and true breeding values was used as the accuracy of a model. The accuracies of genomic estimated breeding values were calculated in two approaches. In the first approach, training animals were first divided into five groups from which in turn, four groups were used to estimate marker effects and the left out group used to calculate accuracies. In the second approach, in order to investigate the persistency of accuracy over generations, estimated marker effects based on animals in reference population, were used repeatedly for measuring the accuracies in the candidate animals (candidate populations) from generation 10 to 15. Unbiasedness of genomic predictions was measured by the regression of true breeding values on estimated genomic breeding values. This regression does not deviate largely from one if the prediction is unbiased.

## Results

Table 1 illustrates the average number of SNP markers and retained PCs which explain 0.999 of the original variance. Although we considered a non-strict criterion for retaining PCs, the number of PCs is nearly half of the number of SNPs. This is the ability of PCA in reducing the variables without considerable loss of variance. Dimauro et al. (2011), selected a strict criteria for retaining PCs and reported that 300 and 700 PCs explain 85 and 95% of the original variance, respectively.

**Table 1:** Average number of SNPs and PCs after quality control, over 10 replicates.

	**105 QTL**	**60 QTL**	**15 QTL**
SNP	2868.4 ± 11.64	2871.1 ± 19.81	2865.8 ± 26
PC	1583.7 ± 7.55	1595.7 ± 15.56	1587 ± 21.97

**Figure 1:**
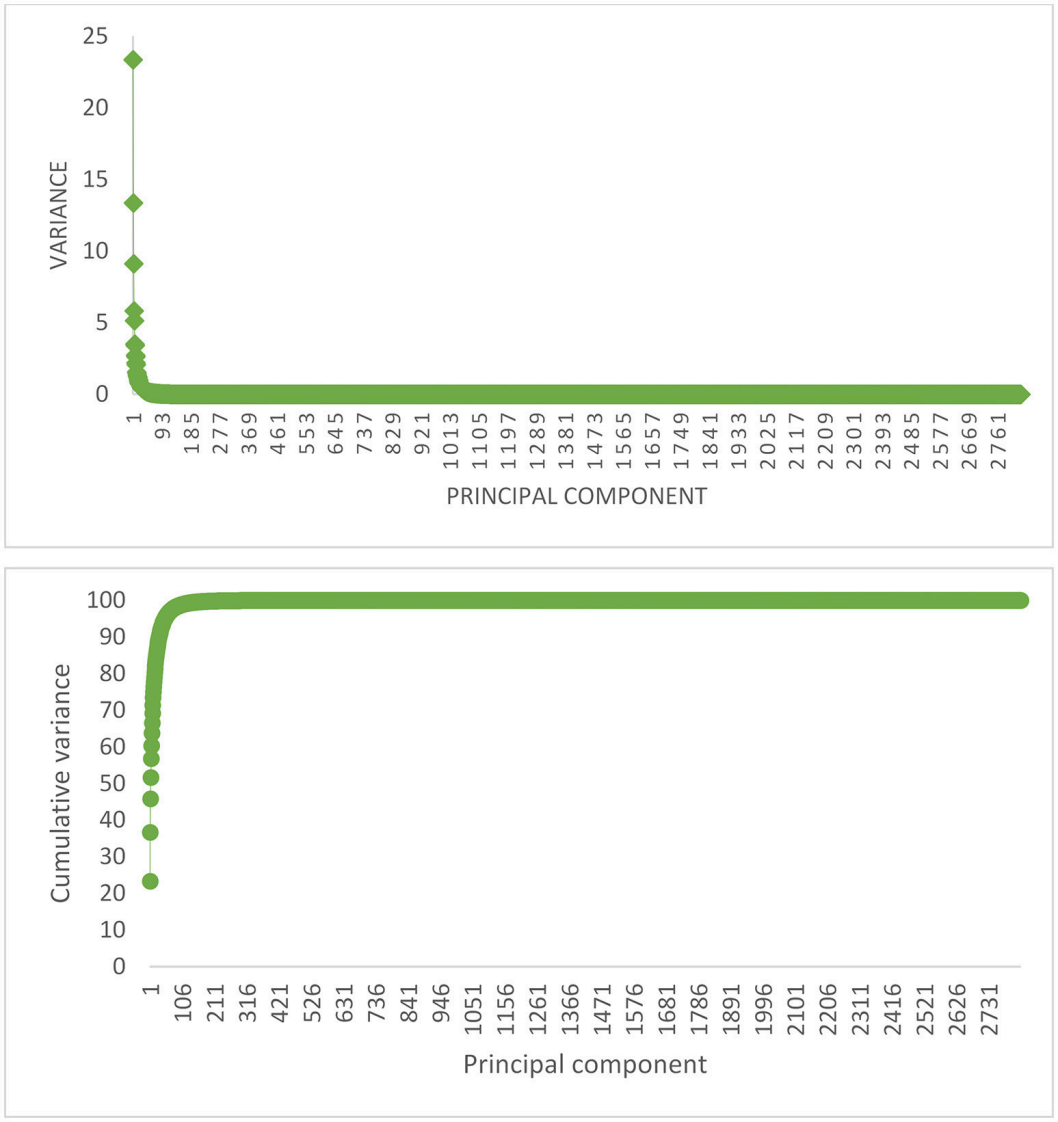
The proportion of variance (%) accounted for by each PC **(Top)**, and the cumulative variance of successive PCs **(Bottom)** for replicate 1 of the scenario with 105 QTL.

The percentage of explained variance by each PC, and also the cumulative variance of PCs for replicate 1 in scenario with 105 QTL is shown in Figure 1, as an example. The first five and 100 PCs are adequate for explaining 60% and 90% of the original variance, respectively. The curve of cumulative variance reached a plateau around 200th PC. In agreement with previous findings on simulated data, PCA has been able to efficiently reduce the size of predictors. Since, a small amount of variance will be explained by each PC after plateau, a large number of PCs must be included in the model to capture a relatively small variance; in this study, about 1,400 PCs after plateau explain less than 1% of the original variance. These results highlight that PC analysis can compress the total variation in a smaller set of variables.

Cross validation accuracies of genomic predictions obtained using SNP/PC based models are shown in Table 2. On average, the accuracies were highest in 105 QTL senario. Accuracy of genomic predictions clearly declined with decreasing QTL from 105 to 15 in all eight methods. As expected, the accuracy of BayesA and BayesB increased with decreasing number of QTLs, and at 15 QTL outperformed Bayes-Ridge model. Previous studies have reported that a BLUP mixed model, assuming equal variance for all SNP, perform as well as variable selection models for most traits in dairy cattle (Hayes et al., 2009; VanRaden et al., 2009), but in traits controled with major genes such as fat percentage, variable selection models are superior over BLUP models (Cole et al., 2009; Legarra et al., 2011). Across all senarios, Ridge-R in SNP based models, and PCR-Eigen in PC based models had lowest accuracies.

**Table 2:** Pearson correlations between predicted genomic breeding values and true breeding values for different methods with five-fold cross validation in training populations.

**QTL**	**Ridge-R**	**Bayes-Ridge|**	**BayesA**	**BayesB**	**PCR-Normal**	**PCR-t**	**PCR-Lasso**	**PCR-Eigen**
105	0.658 ± 0.007	0.671 ± 0.008	0.664 ± 0.009	0.667 ± 0.009	0.664 ± 0.008	0.662 ± 0.009	0.653 ± 0.009	0.641 ± 0.009
60	0.643 ± 0.01	0.654 ± 0.01	0.648 ± 0.01	0.653 ± 0.01	0.651 ± 0.01	0.646 ± 0.01	0.643 ± 0.01	0.625 ± 0.01
15	0.542 ± 0.02	0.553 ± 0.02	0.556 ± 0.02	0.565 ± 0.02	0.551 ± 0.02	0.548 ± 0.02	0.536 ± 0.02	0.504 ± 0.02

*Ridge-R, Ridge regression-BLUP; Bayes-Ridge, Bayesian Ridge regression; PCR-Normal, Bayesian principal component regression with normal distribution of effects; PCR-t, Bayesian principal component regression with scaled t distribution of effects; PCR-Lasso, Bayesian principal component regression with double exponential distribution of effects; PCR-Eigen, Principal component regression-BLUP with eigenvalues as prior variance of effects*.

In all senarios, the performance of PCR-Normal was better than the other three PC based models but the diffrences of PCR-Normal and PCR-t were negligible. Macciotta et al. (2010), investigated the accuracy of PC based estimated breeding values differently. They sequentially added PCs to a PC-BLUP model to reach the highest accuracy and found that the accuracy increased up to a plateau at PC 250 to 300. Retaining more PCs, in their study resulted in no increased accuracy.

In scenario with 105 QTL, the accuracy of Bayes-Ridge, 0.671, was similar to the accuracy of PCR-Normal, that was 0.664, while, in the latter, the size of predictors was nearly half. That is a huge reduction in pridictor variables without any loss of prediction accuracy. In this senario, accuracy of PCR-Normal is exactly similar to the accuracy of BayesA. In 60 QTL senario this two models yielded similar accuracies (0.654 vs. 0.651). This is also true in the case of BayesA and PCR-t, both using the same prior for unknown parameters but the former for SNPs and the later for PC scores. BayesB had the highest accuracy in 15 QTL senario, 0.556, which is only 0.014 higher than the accuracy obtained with PCR-Normal, but 0.052 higher than PCR-Eigen which assumes predictor variances are fixed quantities scaled proportional to their eigenvalues.

A necessary condition for unbiased genomic prediction is that the regression coefficient of true breeding values on genomic prediction is close to 1. Compared with the BLUP models (Ridge-R and PCR-Eigen), the bias in Bayesian models was reduced (Table 3). PCR-Eigen overestimated the genomic breeding values with a regression coefficient of less than 1. In a simulation study by Macciotta et al. (2010) with eigenvalues as prior variance the regression slope was 0.76, and with a single prior variance for PCs it was 0.69. In a simulation study with PCs extracted from different marker densities assuming a single PC variance, regression slopes varied from 0.65 to 0.695 (Solberg et al., 2009a). The data simulated in these studies were different but the methods were comparable to our PCR-Eigen. In contrast to the models with a fixed variance for predictors, Bayesian PC models produced unbiased predictions (Table 3). The unbiased models in 105 QTL scenario were Bayes-Ridge and PCR-Normal and in 60 QTL scenario were BayesA, followed by Bayes-Ridge and PCR-Normal. PCR-t led to unbiased estimated genomic breeding values in 15 QTL scenario.

**Table 3:** Intercept and regression coefficient of true breeding value on predicted genomic breeding value and coefficient of determination for different estimation methods for 5-fold cross validation in training population.

		**Ridge-R**	**Bayes-Ridge**	**BayesA**	**BayesB**	**PCR-Normal**	**PCR-t**	**PCR-Lasso**	**PCR-Eigen**
105	b0	0.88 ± 0.03	0.86 ± 0.04	0.88 ± 0.03	0.86 ± 0.03	0.83 ± 0.04	0.85 ± 0.04	0.85 ± 0.04	0.66 ± 0.04
	b1	1.22 ± 0.04	1.002 ± 0.04	1.05 ± 0.04	1.05 ± 0.05	1.004 ± 0.04	1.09 ± 0.07	1.2 ± 0.08	0.76 ± 0.03
	R^2^	0.48 ± 0.02	0.49 ± 0.02	0.50 ± 0.02	0.50 ± 0.03	0.49 ± 0.02	0.49 ± 0.03	0.48 ± 0.03	0.46 ± 0.02
60	b0	0.85 ± 0.05	0.84 ± 0.05	0.83 ± 0.06	0.84 ± 0.05	0.78 ± 0.05	0.79 ± 0.05	0.81 ± 0.05	0.63 ± 0.05
	b1	1.141 ± 0.05	0.964 ± 0.03	1.007 ± 0.04	1.09 ± 0.05	0.962 ± 0.03	1.05 ± 0.05	1.09 ± 0.05	0.706 ± 0.03
	R^2^	0.46 ± 0.03	0.47 ± 0.03	0.48 ± 0.03	0.48 ± 0.04	0.47 ± 0.03	0.48 ± 0.04	0.46 ± 0.03	0.43 ± 0.03
15	b0	0.94 ± 0.08	0.92 ± 0.08	0.92 ± 0.08	0.90 ± 0.07	0.87 ± 0.08	0.89 ± 0.08	0.90 ± 0.08	0.79 ± 0.1
	b1	0.97 ± 0.1	0.85 ± 0.07	1.02 ± 0.1	0.93 ± 0.06	0.85 ± 0.07	1.007 ± 0.09	1.08 ± 0.1	0.58 ± 0.07
	R^2^	0.35 ± 0.04	0.36 ± 0.05	0.36 ± 0.05	0.38 ± 0.05	0.36 ± 0.05	0.36 ± 0.05	0.35 ± 0.05	0.30 ± 0.05

*b0, Intercept; b1, regression coefficient; R^2^, determination coefficient*.

**Figure 2:**
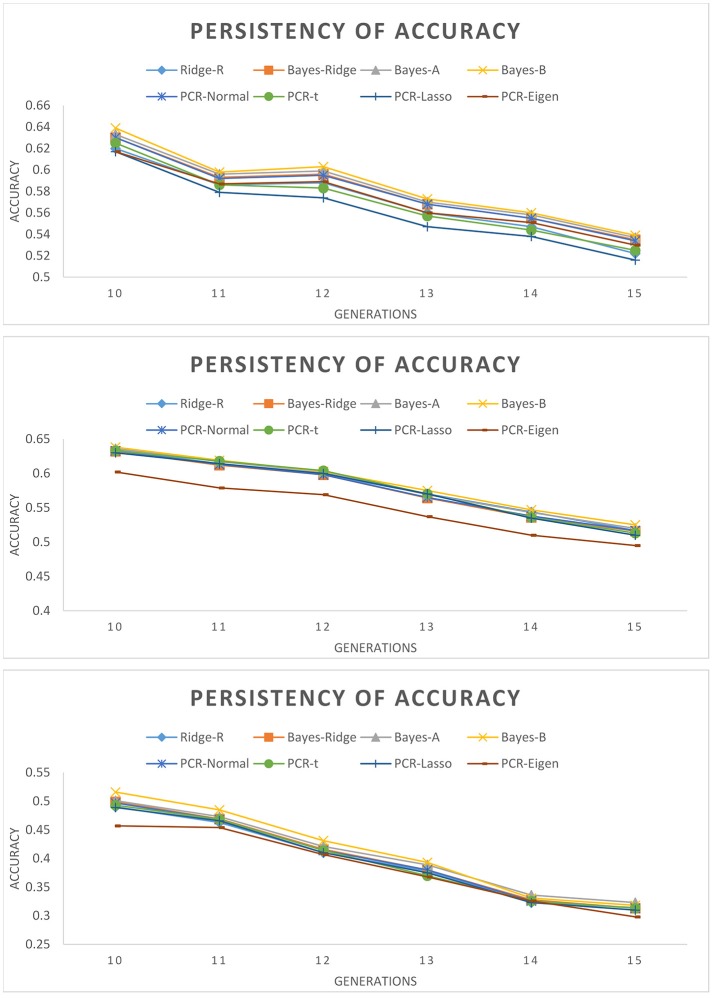
Persistency of accuracy across validation generations measured as correlation between true and estimated genomic breeding values with different estimation methods. **(Top):** 105 QTL; **(Middle):** 60 QTL; **(Bottom):** 15 QTL.

Figure 2 depicts the persistency of selection accuracy over six generations of selection candidates using SNP/PC based models. Accuracies decreased as the number of QTL decreased and as generation increased. This figure shows the marginal differences between SNP based and PC based models for different number of QTL, such that it is difficult to determine which model outperforms the others over the generations. The superiority of Bayesian PCR models over PCR-Eigen is more evident in scenario with 60 QTL followed by 15 QTL.

**Figure 3:**
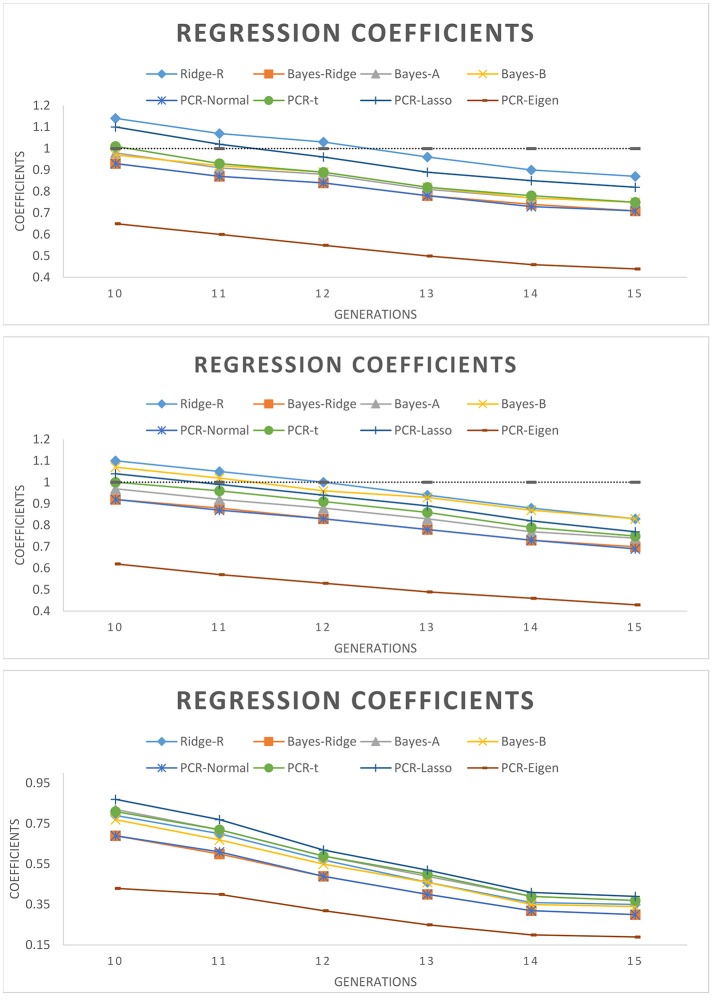
Regression coefficient of true breeding values on estimated genomic breeding values for different generations of selection candidates. **(Top):** 105 QTL; **(Middle):** 60 QTL; **(Bottom):** 15 QTL.

Figure 3 shows the regression coefficients of true breeding values on estimated breeding values over six generations. Across all models, absolute values of regression coefficients decreased as generation increased. PCR-Lasso had an inflated regression slope in the training populations of 105 QTL (b1 = 1.2) and 60 QTL (b1 = 1.09) scenarios, but in generations 10, 11 and even 12 the slope was around 1. PCR-Eigen, consistently overpredicted breeding values such that the regression slope at generation 15 in 15 QTL scenario fell down to 0.19.

## Discussions

Genomic prediction faces a statistical challenge of smaller observations than marker data. Some research in this decade has focused on this challenge and several solutions have been proposed. VanRaden et al. (2009) compared a 40K SNP set with two 20K and 10K subsets that were obtained by keeping every other or every fourth SNP sequentially across genome, respectively, and reported more accurate predictions using 40K SNP panels. The reduction of predictor variables by selecting subsets of SNPs that were evenly spaced or based on their relevance to the trait was investigated by Vazquez et al. (2010). They reported that the accuracy of genomic prediction substantially decreased with subsetting SNPs. Moser et al. (2009) compared several methods to predict genomic breeding values and showed that least squares regression which exploits a reduced subset of selected SNP consistently had lower accuracy and a larger bias of prediction than the other methods using all SNP. Weigel et al. (2009) sorted markers based on magnitude of the estimated marker effects and included only those with the largest effects in the model, but accuracies always declined with subsetting SNPs. In all methods mentioned, eliminating some SNPs produced lower accuracies, while in the genomic prediction reducing dimension of model is advantageous provided that accuracy does not drop considerably. Compared to other subset selection of variables, the multivariate reduction via PCA has the advantage that no marker is discarded, while a smaller set of uncorrelated predictors preserve as much of the variation present in the original markers as possible.

With huge numbers of dense SNPs, the multicollinearity problem due to linkage disequilibrium is unavoidable (Long et al., 2011). Solberg et al. (2009a) employed partial least squares regression (PLSR) and PCA to reduce the dimensionality and showed that when marker density is low, the accuracy of both methods is comparable with BayesB, but with denser markers, BayesB outperforms PLSR and PCA. They concluded that reduction in computational complexity via multivariate methods did not counterbalance their lower accuracy compared with BayesB. Accuracies of genomic predictions obtained using PCR and G-BLUP models was also investigated by Dadousis et al. (2014), who reported across test datasets and traits, G-BLUP outperformed the PCR model. However, in the present study Bayesian estimation of effects and variances of PC scores led to accuracies similar to BayesB and better accuracies than PCR-Eigen where PC variances were proportional to the eigenvalues. Three Bayesian PCR methods performed the same but considering parsimony PCR-Normal with a single variance parameter for PCs is preferred in practice. The performance of models characterized by different prior specifications showed negligible differences in this study. However, it can be the case that the differences in performance of these PCR methods become more visible under broader differences in genetic architectures of the traits.

The persistence of the accuracy of genomic prediction over generations depends largely on the extent of LD and the ability of statistical methods to exploit LD information. BayesB exploits LD information considerably better than Bayesian ridge regression and thus is expected to produce stable accuracy (Habier et al., 2007). Recombination between markers and QTL over generations breaks down linkage disequilibrium and reduces the accuracy of selection. Depending on the cost of genotyping and the number of markers genomic selection programs will be more cost effective if the estimated marker effects could be used over multiple generations (Solberg et al., 2009b). In this study, there were little differences between Bayesian SNP based methods and Bayesian PC based methods in persistency of accuracies across scenarios where BayesB was slightly better than others. Habier et al. (2010) reported that the accuracy of GEBVs decayed over generations but this decay in the accuracy was less in BayesB compared to G-BLUP.

In all scenarios, accuracy of GEBV increased with assuming a prior density for effects and variances of PC scores instead of specifying predefined weights for the PCs; i.e., PCR-Eigen. Although, we can consider the heterogeneous structure of variance by specifying eigenvalues as prior variance for PC scores, but assumption of fixed quantity limits the ability of this proposal. In Bayesian setting, assigning an informative prior density for PC variance(s) combined with information brought by the data leads to more robust estimation of PC effects that in turn leads to greater accuracy. The decay of accuracy in selection candidates over generations tended to be smaller for developed Bayesian PCR; it is even evident when QTL number was smaller.

## Conclusion

The present study assessed the performance of PC based models as a dimensionality reduction method, in comparison to commonly used SNP based models. Accuracies of genomic predictions using prior knowledge of PC effects and variances in a Bayesian hierarchical framework were considerably higher compared to specifying fixed PC variances proportional to eigenvalues. Bayesian PC based models and SNP based models performed similarly at different QTL densities, while the number of predictors in PC models was nearly half of the number of SNPs. Reducing dependency among predictors due to LD as well as dimension reduction via conforming PCs, and then Bayesian updating of PC variance(s) can potentially improve prediction accuracies. Finally, developed methods in this study are recommended according to the ease of implementation and good statistical properties for analysis of correlated high dimensional datasets that are becoming available. These results when confirmed on real data sets, will support the use of Bayesian PCR in genomic predictions.

## Author contributions

SH-V designed and ran the analyses, interpreted the results, and wrote the manuscript. MS assisted with the study design, interpretation of results, and critically contributed to the manuscript. HM and MT helped in the interpretation of results and edited the drafted manuscript. All authors read and approved the final manuscript.

### Conflict of interest statement

The authors declare that the research was conducted in the absence of any commercial or financial relationships that could be construed as a potential conflict of interest.
